# Short-term resistance training enhances functional and physiological markers in older women: implications for biomechanical and health interventions in aging

**DOI:** 10.3389/fpubh.2025.1630525

**Published:** 2025-07-28

**Authors:** Mine Akkuş Uçar, Coşkun Yılmaz, Hakan Hüseyin Soylu, Barış Sarıakçalı, Cemalettin Budak, Korhan Kavuran, Mehmet Vakif Durmuşoğlu, Levent Ceylan

**Affiliations:** ^1^Faculty of Sport Sciences, Mardin Artuklu University, Mardin, Türkiye; ^2^Gümüşhane University, Gümüşhane, Türkiye; ^3^Radiology Department, Gumushane Provincial Health Directorate, Kelkit State Hospital, Gümüşhane, Türkiye; ^4^Department of Internal Medicine, Faculty of Medicine, Sivas Cumhuriyet University, Çanakkale, Türkiye; ^5^Faculty of Sport Sciences, Erzincan Binali Yıldırım University, Erzincan, Türkiye; ^6^Faculty of Sport Sciences, Bitlis Eren University, Bitlis, Türkiye; ^7^Ministry of Youth and Sports, Ankara, Türkiye; ^8^Faculty of Sport Sciences, Hitit University, Çorum, Türkiye

**Keywords:** aging, exercise, gait, health, resistance training, older women

## Abstract

**Background:**

The impact of resistance training extends beyond the enhancement of muscle strength, encompassing improvements in physical performance, postural stability, and overall functional capacity. This study aimed to investigate the effects of a 4-week resistance training program on functional capacity, respiratory muscle strength, diaphragm thickness, and liver density in healthy older women.

**Methods:**

The study included 30 healthy women aged 60–80 years and was designed as a randomized controlled experimental trial. Participants were randomly assigned to a resistance training (RT) group or a control (CON) group. Each participant attended the laboratory on three occasions: during the initial visit, the study procedures were explained; the second visit involved baseline assessments (6MWT, diaphragm thickness and liver fat via ultrasound, and respiratory muscle strength); and final measurements were conducted after the 4-week training programme during the third visit.

**Results:**

When pre- and post-training measurements were compared, the resistance training (RT) group demonstrated a significantly greater improvement (8.02%) in the Six-Minute Walk Test (6MWT) distance compared to the control group (*p* < 0.001). In terms of diaphragm thickness (DT), the RT group showed a 16.66% increase, whereas the control group exhibited a 1.13% decrease (*p* < 0.001). Maximal inspiratory pressure (MIP) increased by 12.30% in the RT group, while it decreased by 7.25% in the control group, indicating a 19.55% greater improvement in the RT group (*p* < 0.001). Regarding maximal expiratory pressure (MEP), a significant improvement of 21.48% was observed in the RT group, whereas a 0.75% decrease was recorded in the control group, resulting in a 22.23% greater enhancement in the RT group (*p* < 0.001). Finally, liver density (LD) increased by 12.30% in the RT group, while it decreased by 7.25% in the control group (*p* < 0.001).

**Conclusion:**

As a result of resistance training, greater improvement was observed in functional capacity, respiratory muscle strength, diaphragm thickness and liver density in the training group compared to the control group.

## 1 Introduction

The contemporary lifestyle engenders a plethora of challenges that bear a strong resemblance to those experienced by preceding generations. However, it is important to recognise that the nature of these challenges has undergone a significant transformation over time. This change has also affected the way modern people protect their health, and the main risk factors related to health have evolved (1). While the importance of health protection for the maintenance of quality of life is widely acknowledged, the factors affecting this process have shown a great change over time. In the contemporary context, the identification of determinants of health and the monitoring of their impact over time has become imperative for individuals to develop health protection strategies (2, 3).

In contemporary societies, physical activity and fitness have emerged as pivotal contributors to overall health and well-being. Adequate levels of physical activity have been shown to be positively correlated with improved biomarkers and quality of life (4). Research in this field has emphasised the beneficial effects of physical fitness on general health. In this context, physical activity is recognised as a significant strategy for maintaining health and increasing functional capacity, particularly in ageing individuals. As the ageing process progresses, a range of health problems become increasingly prevalent, including sarcopenia (loss of muscle mass), decreased muscle strength and reduced functional capacity (1, 2).

Sarcopenia is frequently associated with cardiometabolic health-related conditions and shares common risk factors such as increasing age, physical inactivity, chronic inflammation and malnutrition (5–7). Physiological changes associated with these conditions include biological features that overlap with sarcopenia, such as reduced skeletal muscle fibre count, type II fibre atrophy, motor unit loss, increased fat infiltration, decreased capillarisation, chronic inflammation, increased oxidative stress levels, and decreased insulin sensitivity (8–10). As demonstrated in the extant literature, these changes can be mitigated to a considerable extent by regular physical activity and exercise.

Aging is of great importance in terms of the effects of physical activity on individuals. Such effects include the maintenance of functional independence, physical capacity and reduced disease risk (11). In this process, parameters such as respiratory muscle strength and diaphragm thickness play a significant role in determining respiratory functions and general physical capacity. While respiratory muscles undergo atrophy with age, the thickness of the diaphragm is a significant biomarker of respiratory capacity (4). Furthermore, an increased liver fat percentage has been demonstrated to directly impact metabolic health and insulin resistance. This suggests that the accumulation of liver fat in older individuals may be a contributing factor to the development of cardiometabolic diseases (2).

Resistance training (RT) is widely recognized as the most effective intervention for mitigating age-related declines in muscle mass, strength, and physical function (12–15). In particular, progressive RT has been shown to be a safe and effective strategy for preventing and even reversing sarcopenia (16, 17). Beyond improvements in muscle strength, RT in older adults contributes to enhanced respiratory muscle function, increased diaphragm thickness, and reductions in hepatic fat content (18, 19). Functional gains such as better balance, reduced fall risk, and improved neuromuscular activity have also been well documented (20, 21). However, the benefits of RT are dependent on dynamic and individualized exercise prescriptions that evolve over time (22). Key factors such as training intensity, movement velocity, and professional supervision play a critical role in maximizing the effectiveness of RT in the aging population (23, 24).

The hypothesis of the present study is that resistance training will affect diaphragm muscle thickness, respiratory muscle strength, liver fat ratio and 6-min walk test (6MWT) performance in older women. In consideration of this hypothesis, the present study sought to examine the impact of resistance training on diaphragm thickness, respiratory muscle strength, liver fat percentage and 6-min walk test (6MWT) performance in older women. The evaluation of the relationship between these parameters and the positive effects of resistance training on the overall health of older women will be an important step in the development of new strategies to improve quality of life.

## 2 Materials and methods

### 2.1 Participants

The study comprised 30 healthy women aged 60–80 years. The study was designed as a randomised controlled experimental study. Participants were randomly assigned to two separate groups: The subjects were divided into two groups: the RT group and the CON group. The GPower 3.1 programme was utilised to ascertain the requisite number of participants. The findings of the power analysis sampling study indicated that the study could be completed with 12 subjects in each group (effect size: 0.80; actual power: 0.89). In order to circumvent the potential for complications, a total of 15 participants were included in each group. The numbers from 1 to 30 were randomly assigned to the two groups by a computerised programme in order to ascertain which group the subjects forming the sample would be included in.^1^ In the study, all participants in the RT group underwent the same training programme to rule out contralateral effects (25). The control group continued their daily routines without training. Individuals failing to meet the subsequent criteria were excluded from the study: The patient is between 60 and 80 years of age, has a chronic disease, and has undergone surgery within the last year. Prior to the commencement of the study, all participants were requested to provide both verbal and written consent.

### 2.2 Experimental design

**Figure 1:**
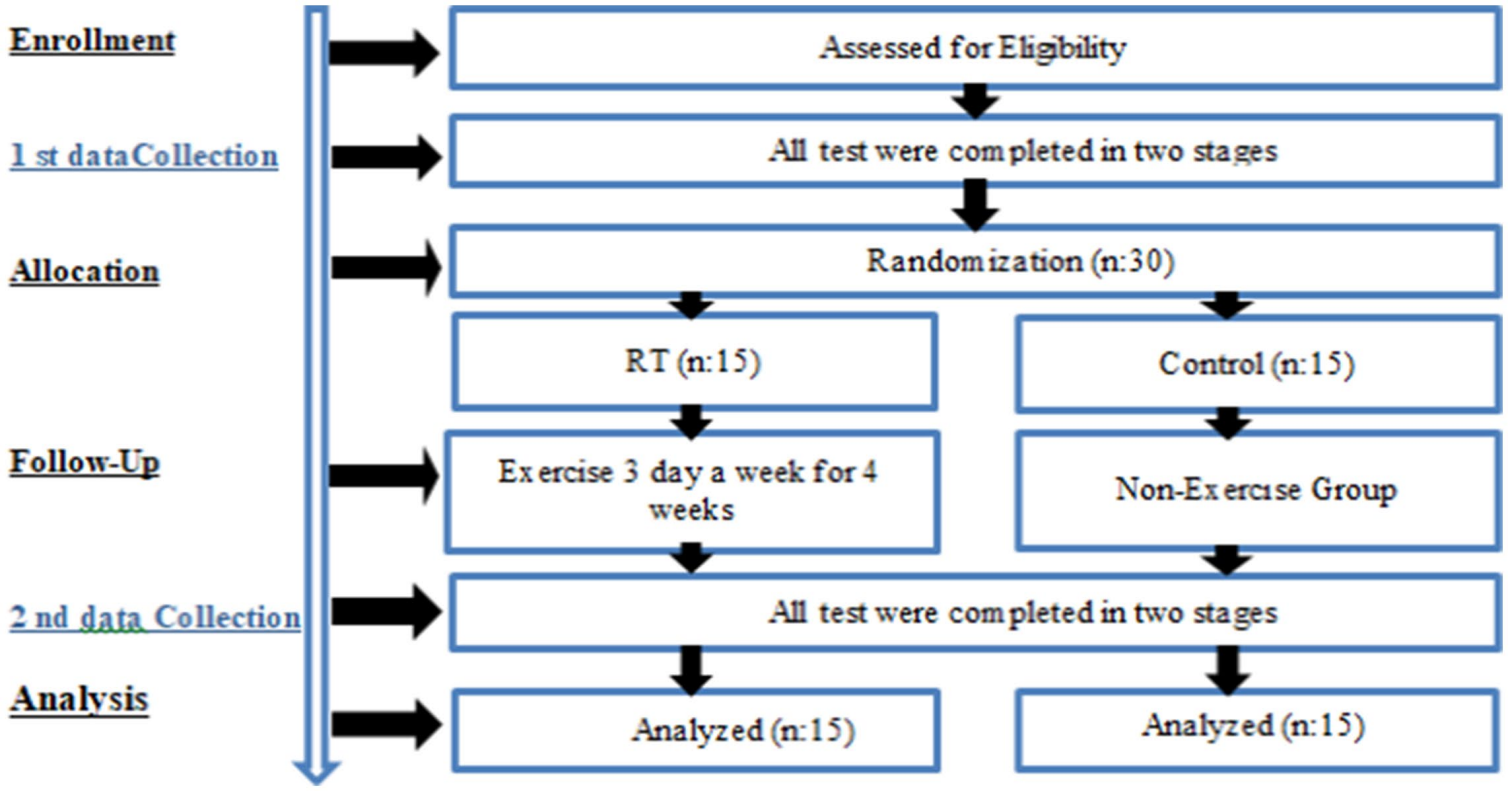
Experimental design.

Female participants within the age range of 60–80 years were requested to visit the laboratory environment on three separate occasions. During the initial visit, the experimental procedures were introduced and tested. Prior to commencing the experiment, each participant was provided with a comprehensive explanation of the RT procedure. On the second occasion, a week later, a series of pre-training measurements were taken and recorded. These comprised the 6-min walk test (6MWT), ultrasonography, diaphragm thickness and fatty liver ratio with a radiologist, and finally respiratory muscle strength tests. At the conclusion of the 4-week resistance training programme, final measurements were obtained during the third and final visit (see Figure 1).

### 2.3 Body composition measurement

Gaia 359 Plus Body-pass bioelectrical impedance analyser was used to measure the body composition of the athletes visiting the laboratory of Gümüşhane University. This device uses a measurement method that generates and calculates information about the tissue according to the type of resistance encountered by low electrical currents as they move between body tissues. Gaia 359 Plus BodyPass was used to determine the height, body weight and body mass index (BMI) of the subjects. Subjects were instructed to stand on the analyser with the entire soles of their bare feet in contact and to remove all outer clothing, including t-shirts and shorts. Subjects were instructed to remove all metal objects before the start of the measurement.

### 2.4 Six-minute walk test (6MWT) protocol

All participants were provided with instructions for the Six-Minute Walk Test (6MWT) by an exercise physiologist. Participants were asked to walk as fast as possible at a comfortable pace for 6 min along a previously measured straight path. During the test, the evaluator recorded the time and provided standard encouragement at each minute interval. The evaluator also counted the number of laps completed by each participant. At the sixth minute, participants stopped at their current location on the path, and the evaluator measured the total distance walked for the final lap. The evaluator then calculated and recorded the total distance walked during the 6-min period (26).

### 2.5 Liver density analysis

In this study, data obtained from female subjects aged 60–80 years who underwent CT scanning of the thorax or abdomen for any clinical indication were used. Imaging was performed with a Siemens Somatom Definition AS 128 model computed tomography device. Participants were given breathing exercises before imaging. On the day of the imaging, the participants were placed on the device table in the supine position; after the device settings were completed, they were asked to take a deep breath and hold their breath just before the imaging started (27). Images were acquired in the inspiratory phase. In the acquired CT images, liver thickness was measured at a point with a relatively homogeneous thickness at the level of the liver dome in the posteromedial region of the right hemidiaphragm and the data were recorded. In addition, in the same participants, the density was measured from a homogeneous parenchymal area without obvious lesions in the segment 8 region of the liver, adjacent to the middle hepatic vein, and the relevant data were recorded. As a result of the recorded data, the device automatically calculated and recorded the liver fat content. A low value of liver fat content <33 represents more liver fat (28).

### 2.6 Maximal inspiratory (MIP) and expiratory (MEP) pressure measurements

MIP and MEP were measured with a portable handheld oral respiratory pressure gauge (MicroRPM, CareFusion Micro Medical, Kent, UK) according to the guidelines of the American Thoracic Society and the European Respiratory Society (29). After securing the appropriate filters and holders, the nasal airway was closed with a clip. The mouthpiece assembly had a 1 mm hole to prevent glandular closure and minimise the contribution of the cheek muscles during inspiratory efforts. Inspiratory and expiratory manoeuvres were performed in a seated position, with MIP and MEP measurements initiated from residual volume and total lung capacity, respectively, and continuing for a minimum of 1 s. Each participant completed three repetitions, with a one-minute interval allocated between each set to allow for recovery. The mean values were recorded for all the data relating to MIP and MEP (57).

### 2.7 Diaphragm thickness (DT) measurement

Diaphragm muscle thickness measurements were performed by a radiologist experienced in musculoskeletal ultrasonography using a Philips Affiniti 70G ultrasonography device (Philips Healthcare, Bothell, WA, USA) and a 5 cm wide linear transducer probe at a frequency of 12 MHz. All measurements were performed on the right hemidiaphragm with the participants supine and relaxed. The transducer was placed on the mid-axillary line on the right side in the coronal plane, allowing visualisation through the liver window. Diaphragm thickness was measured at the apposition site of the right haemidiaphragm. The intercostal space between the 8th and 9th costae was determined to perform measurements during the maximal expiratory phase. Using this interval, the optimal diaphragm image was obtained through the liver window (58). For measurement during maximum inspiration, the intercostal space between the 10th and 11th ribs was used to determine the optimal imaging position. Ultrasonographic measurements were performed in two stages. In the first stage, the participant was asked to perform maximum expiration by exhaling as deeply as possible and then hold his/her breath. At this time, the diaphragm muscle was visualised and the muscle thickness was measured. In the second stage, the participant was asked to breathe maximally and hold his/her breath again; thus, the measurement was performed in the inspiratory phase. During the diaphragm thickness measurements, only the data of the muscle tissue were taken into account; echogenic lines formed by the pleura and peritoneum were not included in the measurement. Measurements were repeated three times for each phase and the average of the obtained values was used in the analyses (59).

### 2.8 Resistance training program and protocols

The resistance training (RT) program and protocols were developed in accordance with the recommendations of The American College of Sports Medicine’s (ACSM) to promote optimal athletic development. Training programs for RT group were designed and supervised by an experienced senior coach. The training sessions were conducted three times per week, with each session lasting approximately 30–40 min. Rest intervals between sets were set at 30–60 s. The training intensity was maintained at a moderate level, allowing participants to exercise without significant strain and while being able to engage in conversation (17, 30). Perceived exertion was monitored 1–2 times per week using the Rating of Perceived Exertion (RPE) scale (1–10), targeting a range of approximately 4–6 (Table 1). Prior to each training session, a standardized 10-min dynamic warm-up routine was performed to enhance joint mobility, increase core body temperature, and prepare the cardiovascular and musculoskeletal systems for exercise. The warm-up began with a 4-min low-intensity walk aimed at gradually elevating heart rate. This was followed by dynamic mobility exercises, including 10 repetitions of forward and backward shoulder circles, and gentle cervical movements—flexion, extension, and lateral flexion—to support neck mobility. To stimulate upper body circulation, aerobic arm movements (swinging the arms forward and sideways) were performed for 2 min. This was followed by 2 min of on-the-spot marching with light knee lifts to activate the lower extremity muscles. The warm-up concluded with 2 min of alternating heel-to-glute movements to activate the hamstrings. All warm-up exercises were performed at a light to moderate intensity, in a slow, controlled, and pain-free manner. Participants were instructed to use support from a chair or wall if needed to ensure safety (17, 31, 32).

**Table 1:** Weekly programme.

Days	Exercise	Sets × Reps	Description
Day 1 Monday	Chair Sit-to-Stand (Squat)	3 × 12	At knee level, slow and controlled
	Glute Bridge	3 × 15	Lying on floor or bed
	Standing Hip Abduction (with Resistance Band)	2 × 12/leg	Balance support may be required
	Core: Seated Knee Raises	2 × 10	While seated, pull one knee toward the chest
	Respiratory: Pursed-Lip Breathing +Deep Diaphragmatic Breathing	2 × 1 min	Inhale through the nose, exhale slowly through pursed lips
Day 2 Wednesday	Wall Push-Up	3 × 10–12	Body straight, hands at shoulder height
	Seated Row with Resistance Band	3 × 12	Band can be fixed to chair
	Lateral Shoulder Raise (with Dumbbell or Water Bottle)	2 × 12	Lift arms to shoulder height
	Biceps Curl	2 × 12	With light dumbbells
	Respiratory: MIP-MEP Exercises	3 × 5 breaths	Using balloon inflation
Day 3 (Friday)	Step-Up/Down on Stairs	3 × 6–10 steps	Slow and careful, use support if needed
	Mini Squat + Ball Chest Press	2 × 10	Controlled chest press using a ball against the wall
	Single-Leg Balance (Standing)	2 × 20 s/leg	Use wall support if necessary
	Hip Stretch + Trunk Rotation	2 × 8	Slow and rhythmic movement
	Respiratory: Diaphragmatic Breathing + Trunk Movement	2 × 1 min	Hands on abdomen, seated or standing

### 2.9 Statistical analysis

Statistical analyses were performed via SPSS (Version 21.0 for Windows, Chicago, IL, USA) software, with the statistical significance set at 0.05. The Shapiro–Wilk normality test was performed to determine the homogeneity of the sample. Each pre-test and post-test differences were determined by paired comparison test (paired *t*-test), and inter-group differences were determined by one-way analysis of variance with post-test and pre-test difference values. In addition, the effect size in the comparison of paired groups was calculated according to Hedges’ g (33). It was also interpreted as follows: 0–0.19 insignificant, 0.20–0.59 small, 0.6–1.19 medium, 1.20–1.99 large and ≥2.00 very large.

## 3 Results

**Figure 2:**
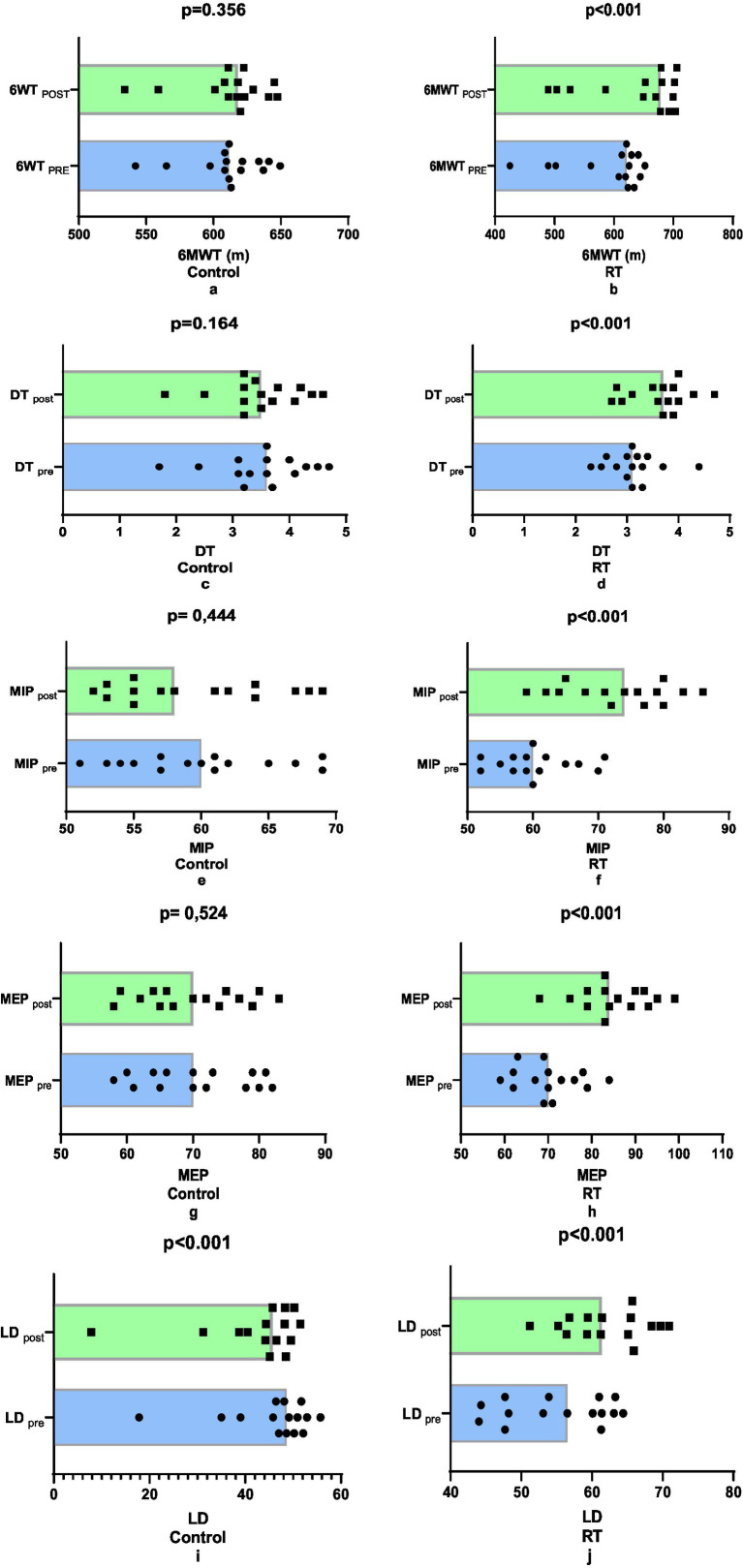
Comparison of pre-post training performance parameters.

A comparison of the 6MWT values before and after training revealed that the RT group demonstrated a significantly higher improvement of 8.02% (*p* < 0.001, Figures 2a,b). This represents a marked increase in comparison to the control group, which exhibited a comparatively minor improvement of 0.19% (*p* = 0.356). In the comparisons made in terms of DT values, it was determined that there was a 17.79% greater improvement in the RT group (%: 16.66, e.s:0.971, *p* < 0.001) compared to the control group (%: −1.13, e.s:0.053, *p* = 0.164) (*p* < 0.001, Figures 2c,d). In the comparison of Maximal Inspiratory Pressure (MIP) values, it was determined that there was a 19.55% more marked improvement in the RT group (%: 12.30, e.s:1.030, *p* < 0.001) compared to the control group (%: −7.25, e.s:0.327, *p* < 0.001) (*p* < 0.001, Figures 2e,f). The Maximal Expiratory Pressure (MEP) data was analysed, revealing a 22.23% greater improvement in the RT group than in the control group (RT: 21.48%, e.s. 1.980, *p* < 0.001; control: −0.75%, e.s. 0.066, *p* = 0.524) (*p* < 0.001, Figures 2g,h). Finally, a comparison of LD values revealed a 19.55% improvement in the RT group (12.30%, e.s. 1.030, *p* < 0.001) compared to the control group (7.25%, e.s. 0.327, *p* < 0.001) (*p* < 0.001, Figures 2i,j) (see Tables 2, 3).

**Table 2:** Descriptive.

	Control (n:15)		RT (n:15)	
	X	SD	X	SD
Age	70.93	5.80	71.8	9.41
Height	164.2	3.32	161.8	4.81
Weight	74.33	4.03	76.6	4.31

**Table 3:** Comparison of pre-post training performance parameters.

Parameters		Control			RT			*p*
		X	SD	*g*	X	SD	*g*	
6MWT	Pre	611.34	27.85	0.042	592.61	67.47	0.677	*p* < 0.001
	Post	612.56	30.34		641.32↑	76.25		
DT	Pre	3.53	0.79	0.053	3.12	0.51	0.971	*p* < 0.001
	Post	3.49	0.72		3.64↑	0.56		
MIP	Pre	60.00	5.68	0.082	60.47	5.79	1.790	*p* < 0.001
	Post	59.53	5.84		73.07↑	8.11		
MEP	Pre	70.60	8,1	0.066	70.13	7.05	1.980	*p* < 0.001
	Post	70.07	7.83		85.2↑	8.13		
LD	Pre	46.02	9.41	0.327	55.33	7.36	1.030	*p* < 0.001
	Post	42.68↓	10.96		62.14↑	5.77		

g, Hedges’ g, ↑,↓; *p* < 0.05; 6MWT, Six-Minute Walk Test; DT, Diaphragm Thickness; LD, Liver Density; MIP, Maximal inspiratory pressure; MEP, Maximal expiratory pressure.

## 4 Discussion

The central hypothesis of this study that resistance training (RT) would affect, respiratory muscle strength, diaphragm thickness, and liver fat percentage in healthy older adult women was confirmed. The findings indicated that the RT group exhibited significant enhancements in both pre- and post-training measurements in comparison to the control group. A notable increase in key parameters was observed in the RT group, with significant rises recorded in the 6 Minute Walk Test (6MWT), Diaphragm Thickness (DT), Maximal Inspiratory Pressure (MIP), Maximal Expiratory Pressure (MEP), and Liver Density (LD), with respective increases of 8.02, 17.79, 19.55, 22.23, and 19.55%.

These results are consistent with the literature reporting that resistance training is effective in increasing respiratory muscle strength and diaphragm thickness in older adult individuals. Özdoğan et al. (34) reported that inspiratory muscle training improved muscle strength, exercise capacity and quality of life in individuals with sarcopenia, while Lee et al. (35) reported that respiratory muscle strength was associated with diaphragm thickness and sarcopenia indices in older adult individuals. Similarly, Flor-Rufino et al. (36) showed that high-intensity resistance training halts age-related decline in respiratory function and increases muscle strength. The present findings support the aforementioned literature and reveal the positive effects of resistance training on the respiratory system.

The study also found a significant increase in liver density, suggesting a reduction in fatty liver tissue as a result of RT. In their 2021 study, Hejazi and Hackett (37) reported that exercise improved liver function in patients with non-alcoholic fatty liver disease (NAFLD). Consequently, the results of this study indicate that RT may also exert beneficial effects on hepatic health.

With respect to functional capacity and quality of life, the 6MWT results underscore the impact of RT in enhancing aerobic capacity and physical endurance. According to Seo et al. (38), the administration of resistance training (RT) has been demonstrated to enhance functional fitness by mitigating age-related intramuscular fat accumulation. Pinto et al. (39) revealed that even short-term strength training improves functional capacity by increasing muscle quality in older women. In accordance with the aforementioned findings, the present study posits that a brief rehabilitation intervention may enhance the physical capacity of older adult individuals.

The findings on quality of life are consistent with the extant literature on the subject. In a 2020 study, Šarabon et al. (40) reported significant effects of resistance training on body composition and functional capacity in older adult individuals. The same research group (41) revealed that flywheel exercises provided greater improvements in various cardiometabolic and musculoskeletal parameters compared to conventional RT. Moreover, meta-analyses conducted by Khodadad Kashi et al. (42) and Hart and Buck (43) support the efficacy of RT as a strategy to enhance quality of life. The present study found that the application of RT led to significant improvements in quality of life. These results are consistent with the findings of Pedersen et al. (44) and Levinger et al. (45), which emphasize the positive effects of RT on psychological health and activities of daily living.

Resistance training (RT) is recognised as a primary strategy for the prevention and management of sarcopenia (16, 17, 31). RT has been demonstrated to offer numerous benefits in terms of increasing muscle mass, muscle strength, endurance, strength and physical function, as well as reducing the risk of associated injuries such as falls and fractures (12–14, 46). These adaptations support the fundamental muscle functions necessary to sustain activities of daily living, particularly among older adults and clinical populations (47–50). Furthermore, a substantial body of research has repeatedly demonstrated the safety, efficacy, and recommendation of RT for both healthy older adults and individuals experiencing diverse disease states (17, 31, 51–56). It has been established that muscle density is more strongly associated with physical performance than muscle size, particularly in women. Consequently, muscle density is likely to result in a more clinically relevant muscle performance than muscle size (49).

In conclusion, this study revealed that resistance training had significant and positive effects on respiratory muscle strength, diaphragm thickness, liver fat percentage, functional capacity and quality of life in healthy older adult women. The findings suggest that RT offers important contributions to not only musculoskeletal but also cardiorespiratory and metabolic health in older adult individuals. In this context, it is suggested that RT should be included in holistic health strategies to support the healthy aging process of older adult individuals.

A paucity of studies has been conducted on the effects of RT on respiratory muscle strength, diaphragm thickness, liver fat percentage, functional capacity and quality of life in older adult women. This finding can be regarded as a significant strength of the present study. The present study is not without its limitations. The number of participants was limited to women who were healthy and aged between 60 and 80 years. The restricted sample size precluded the generalisability of the findings. A larger sample size may provide more accurate data. The assessment process comprised four fundamental assessments: The following methods of assessment were employed: the 6-min walk test (6MWT), the measurement of respiratory muscle strength, the Short Form 12 (SF-12) health survey, and ultrasonography. The temporal limitation of the study, encompassing a mere 4 weeks, precluded the evaluation of long-term outcomes, impeding the discernment of enduring effects. Furthermore, the study did not address potential gender differences that may have affected the results. In future studies, researchers should consider the examination of long-term effects, the consideration of different measurement parameters, and the investigation of a wider range of exercise protocols. This would provide a more comprehensive understanding.

## 5 Conclusion

The central hypothesis of this study, which suggested that resistance training (RT) would affect quality of life, respiratory muscle strength, diaphragm thickness and liver fat percentage in healthy older women, was confirmed. The incorporation of resistance training (RT) into the daily lives of healthy older women was associated with greater improvements in physical function when compared with those who did not engage in such training. In light of these findings, it is recommended that RT be incorporated into the daily lives of healthy older women.

## Data Availability

The original contributions presented in the study are included in the article/supplementary material, further inquiries can be directed to the corresponding author/s.

## Footnotes

^1^ https://www.randomizer.org/
